# XRP44X, an Inhibitor of Ras/Erk Activation of the Transcription Factor Elk3, Inhibits Tumour Growth and Metastasis in Mice

**DOI:** 10.1371/journal.pone.0159531

**Published:** 2016-07-18

**Authors:** Kostyantyn Semenchenko, Christine Wasylyk, Henry Cheung, Yves Tourrette, Peter Maas, Jack A Schalken, Gabri van der Pluijm, Bohdan Wasylyk

**Affiliations:** 1 Institut de Génétique et de Biologie Moléculaire et Cellulaire, Illkirch, France; 2 Centre National de la Recherche Scientifique, UMR7104, Illkirch, France; 3 Institut National de la Santé et de la Recherche Médicale, U964, Illkirch, France; 4 Université de Strasbourg, Illkirch, France; 5 Leiden University Medical Center, Leiden, The Netherlands; 6 SPECS, Kluyverweg 6, 2629 HT Delft, The Netherlands; 7 Radboud University Medical Center, Nijmegen, 6525 GA, The Netherlands; Virginia Commonwealth University, UNITED STATES

## Abstract

Transcription factors have an important role in cancer but are difficult targets for the development of tumour therapies. These factors include the Ets family, and in this study Elk3 that is activated by Ras oncogene /Erk signalling, and is involved in angiogenesis, malignant progression and epithelial-mesenchymal type processes. We previously described the identification and in-vitro characterisation of an inhibitor of Ras / Erk activation of Elk3 that also affects microtubules, XRP44X. We now report an initial characterisation of the effects of XRP44X in-vivo on tumour growth and metastasis in three preclinical models mouse models, subcutaneous xenografts, intra-cardiac injection-bone metastasis and the TRAMP transgenic mouse model of prostate cancer progression. XRP44X inhibits tumour growth and metastasis, with limited toxicity. Tumours from XRP44X-treated animals have decreased expression of genes containing Elk3-like binding motifs in their promoters, Elk3 protein and phosphorylated Elk3, suggesting that perhaps XRP44X acts in part by inhibiting the activity of Elk3. Further studies are now warranted to develop XRP44X for tumour therapy.

## Introduction

Transcription factors are potent drivers of cell transformation, and targeting their activity can be highly effective for tumour therapy [1]. Transcription factors of the Ets family play important role in tumorigenesis [2] and are being used as targets to develop therapies [3]. We showed that the Elk3 member of the Ets family is a potential target for tumour therapy. Elk3 can act as either a repressor of transcription or an activator following phosphorylation by Erk1/2 in response to growth factor activation of the Ras pathway [4]. Elk3 is downregulated by hypoxia and regulates hypoxia-responsive genes [5–7]. Elk3 is involved in angiogenesis [8–12], malignant progression of squamous cell carcinoma [13], cell adhesion, migration and invasion [14–16], and differentiation from progenitor to definitive neural crest cell [17]. We identified an inhibitor of Ras-activated Elk3 transcription-factor activity, XRP44X, in a cell based screen of a small-molecule library [18]. The scaffold of XRP44X is pyrazole, which is considered to be very useful for the development of anticancer agents [19]. XRP44X was found to inhibit fibroblast growth factor 2 (FGF-2)-induced Elk3 phosphorylation by the Ras-Erk signaling at some point in the pathway upstream from Ras. It was also found to bind to the colchicine-binding site of tubulin, to depolymerize microtubules, to stimulate blebbing of the cell membrane and to alter the actin skeleton.

Various classes of tubulin directed agents have been identified. They are considered to be clinically significant because they have a broad spectrum of effects that include cell division, intracellular trafficking, cell signalling, cell migration and angiogenesis [20]. Microtubule-targeting agents can either stabilise or destabilise microtubules [21]. We found that XRP44X and the destabiliser, Combretastin-A4, have similar effects on the cytoskeleton and FGF-2 Ras-Elk3 signalling, and they differ from other classes of agents, and in particular docetaxel, that stabilises microtubules. [18]. At least some of these effects of XRP44X are mediated by c-Jun N-terminal kinase [22]. These in-vitro properties encouraged the investigation of the therapeutic properties of XRP44X in pre-clinical cancer models.

Preclinical models are indispensable in the drug discovery and development even though they are imperfect replicas of human cancers. There are various preclinical tumour models, including traditional ectopic xenografts and genetically engineered tumour models, that represent a portfolio of test systems that are used in a hierarchical manner [23]. We have undertaken an initial evaluation of XRP44X in order to broadly establish its efficacy and toxicity. We used well established models of increasing complexity, including several ectopic xenografts in nude mice that generate metastases (subcutaneous xenografts and cardiac injection of tumour cell lines) and the transgenic adenocarcinoma mouse prostate (TRAMP) model [24] that is amenable to drug testing [25]. We found that XRP44X inhibits tumour growth and metastasis with limited toxicity by a mechanism that involves Elk3. These results encourage further investigation of XRP44X for tumour therapy in humans.

## Materials and Methods

### Cell Lines, Culture Conditions and Growth Curves

The cell lines C6 (ATCC CCL-107) and LL/2 (LLC1) (ATCC CRL-1642) were propagated at 37°C with 5% CO_2_ in complete medium (see the ATCC webpages for compositions, C6, LL/2). All cell lines were tested to ensure they were mycoplasma free.

### Animals

Animal experiments performed in the IGBMC conformed to French laws, including the latest Decree 2013–118 of 1 February 2013 on the protection of animals used for scientific purposes and the Decree of 1 February 2013 on the ethical evaluation and authorization of projects involving the use of animals in experimental procedures. Ethical issues were overseen by Com'Eth, the local ethics committee for animal experimentation. Com'Eth is registered with the Ministry of Research and the National Ethics Committee for Animal Experimentation Thinking under number 17. Animal well-being is considered in the ethical approvals and is supervised and enforced by the Animal Well-being Committee of the IGBMC, in close collaboration with Com’Eth. The Well-being Committee was established following the Directive 2010/63 (decrees and orders of February 1, 2013; Art. R. 214–102; Art. R. 214–103, Art. 4.–I; Art. 9.–in application of R. 214–126). Animal experiments performed in LUMC followed the guidelines established in the Netherlands for the care and use of laboratory animals (DEC 4077).

Humane endpoints were in place, and animals were euthanized to prevent unnecessary suffering, following the general published guidelines [26] and the recommendations of the Ethical Committees. Tumour burden was limited to the minimum required for a valid scientific outcome, and before there were clinical signs that necessitate immediate intervention (see Box 5 in [26]). Animals were monitored daily for general and clinical signs of potential adverse effects (eating, drinking, weight change, persistent hypothermia, discharge, respiration, enlarged lymph nodes or spleen, weakness, paralysis, anaemia, incontinence, diarrhoea, general behaviour and pain). There were no euthanized mice in the xenograft experiments. Several TRAMP mice were euthanized, mainly near the end of the treatment, due the presence of palpable tumours and persistent weight gain. They corresponded to 5 control and 1 XRP44X treated TRAMP mice, 40, 57, 69, 83 and 88 versus 85 days after the start of treatment of the 98 day protocol. These mice were included in the protocol. Two wild-type mice were lost, due to unknown causes.

### Subcutaneous Xenografts in Nude Mice

Male nude mice (BALB/c nu/nu; Charles River, L’Arbresle, France) were housed in ventilated cages under sterile conditions. C6 and LL/2 (LLC1) cells were detached with trypsin, resuspended in PBS and injected subcutaneously in the left flank of 8 week old female BALB/c nu/nu nude mice (200 μl containing 10^5^ C6 or 10^6^ LL/2 (LLC1) cells, 10 mice per cell line). The smaller and larger tumour diameters were measured with callipers every day, and tumour volumes were calculated [V = 4/3 x π x (1/2 x smaller diameter)^2^ x (1/2 x larger diameter)]. After about 6 days, when the tumour volumes were about 30 mm^3^, the mice were injected intraperitoneally every day with 200 μl of XRP44X (0.1 mM XRP44X in 30% DMSO in PBS) or solvent alone (control) for about 25 days. The mice were anaesthetised (100 μl 25% Imalgen 1000, 15% Rompun in 0.9% NaCl), the primary tumours were excised and frozen in dry ice. The mice were then perfused with fixative (4% paraformaldehyde, 3% glutaraldehyde in PBS), organs were removed and immersed in fixative. Similar results were obtained in three separate experiments.

### Metastasis-to-Bone Mouse Model

Male nude mice (BALB/c nu/nu; Charles River, L’Arbresle, France) were housed in individual ventilated cages under sterile conditions Mice were anesthetized before all surgical and analytical procedures. A single-cell suspension of 1 × 10^5^ PC-3M-Pro4/luc or PC-3MPro4/luc2* cells/100 μl PBS was injected into the left cardiac ventricle of 4-week-old BALB/c nu/nu nude male mice. The progression of cancer cell growth was monitored by bioluminescent imaging (BLI) using the IVIS100 Imaging System (Caliper Life- Sciences, Hopkinton, MA). Analyses for each metastatic site were performed after defining a region of interest and quantified with the Living Image software (Caliper LifeSciences). Values were expressed as relative light units (RLU). After the experimental period, the animals were sacrificed, and tibiae and fibulae were dissected, decalcified and the tissues were processed for paraffin embedding and sectioning (5.0 μM sections) for histomorphometrical and immunohistochemical analysis.

### TRAMP Model

TRAMP mice (C57BL/6-Tg(TRAMP)8247Ng/J), were purchased from The Jackson Laboratory [24]. Heterozygous females were maintained on a pure C57BL/6J background by cross-breeding with non-transgenic C57BL/6 males (Charles River Laboratories, France). Transgenic animals were identified by PCR analysis of tail DNA, as described by [27]. The methodology was based on previous studies using TRAMP mice [28–35]. The XRP44X-treatment protocol was approved by the IGBMC/ICS Ethics Committee (N° 2011–009, 2012–110). 15-week-old WT and heterozygous TRAMP male mice were treated with either XRP44X (1.0 mg/kg) or vehicle (DMSO, Sigma-Aldrich, D5879) by intraperitoneal injection at the same time of day, 6 days per week, from 15 to 29 weeks of age, inclusive. The solutions (XRP44X in 30% DMSO in PBS, or 30% DMSO in PBS) were prepared immediately before use and the maximum volume of injection was 100 μL. The numbers of mice per group were: 14 WT vehicle, 12 WT XRP44X, 21 TRAMP vehicle and 21 TRAMP XRP44X. General health was monitored at the time of injection. Animal body weight was recorded twice a week. Injections of XRP44X were stopped 24 hours before necropsy. At necropsy, the animals were weighed and euthanized by carbon dioxide asphyxiation. Animals were dissected and examined for gross pathology, abnormal organ size and presence of metastases. The genitourinary tract, prepared by removing the bladder, seminal vesicles and prostate en bloc and draining the bladder, was weighed. The prostate gland was subsequently dissected for further analysis. Various organs were harvested, including the pelvic lymph nodes, liver, lungs, kidneys and spleen. Tissue samples were placed in 10% v/v phosphate-buffered formalin for 12–24 h, after which the formalin solution was replaced with 70% ethanol (v/v). For routine histopathological evaluation, the tissues were paraffin embedded, sectioned (5.0 μm thickness) and stained with H/E. Organ weights were determined using formalin fixation, ethanol stored organs. Whole blood and serum analyses were performed by the ICS metabolic exploration platform (References: IP00005077, IP0000004829, IP0000004857, IP0000004895, IP0000004915, IP0000004949, IP0000005011). For blood cell analysis, 4–5 days before the end of the XRP44X-treatment protocol, whole blood was taken from periorbital sinuses of animals that had not been treated for 36 hours and was analysed on the same day. For blood biochemical analysis, whole blood was taken by cardiac puncture during necropsy from animals that had not been for 48 hours. Plasma was prepared and stored at -20°C before use.

### Immunohistochemistry

Immunohistochemistry was performed using antibodies against Elk3 (1:1000, peptide affinity purified PAb-95 [36] and Sigma Prestige® HPA001600) and P-Elk3 (1:2500, peptide affinity purified MAb-2F3 [8]) and ABC Vectastain kits (Vector laboratories, PK-6101, PK-6102). Tissue slides were heated on a hot plate (5 min 58°C), cleared with Histosol (2 x 3`min), washed with 100% ethanol (2 x 3 min), 95% ethanol (2 x 3 min), 70% ethanol (2 x 3 min), water (3 min), heated in a pressure cooking in 10mM sodium citrate (5 min 125°C, 20 min cooling in the cooker), washed with PBS-T (5 min), blocked (goat serum in PBS-T, 1 h), incubated with primary Ab (1:1000 in blocking serum in PBS-T, 4°C, 14 h), washed with PBS-T (2 x 5 min), incubated with secondary Ab (in blocking serum in PBS-T, 1 h), washed with PBS-T (2 x 5 min), PBS (2 x 5 min), incubated with ABC reagent in PBS (30 min), washed with PBS (5 min), incubated with DAB (2–10 min), washed with water (1 min), stained with Harris haematoxylin (2 sec), washed with water (30 sec), 70% ethanol (1 min), 100% ethanol 2 x 1 min), and incubated with Histosol (2 x 1 min). Staining was quantified by counting the total numbers of unstained and stained cells in 3–4 representative fields. The numbers counted for the LLC1 xenografts were: control and PAb-95, 158 total, 90 stained; XRP44X and PAb-95, 537 total, 54 stained; control and MAb-2F3, 580 total, 57 stained; XRP44X and MAb-2F3, 540 total and 70 stained. For dorsolateral prostates from mice, the numbers were: wild type PAb-95, 164 total, 47 stained; TRAMP PAb-95, 449 total, 330 stained; TRAMP vehicle MAb-2F3 vs. TRAMP XRP44X an average of 179 stained cells were counted from the selected dorsolateral prostate microscopic fields.

### Statistical Analysis

Fisher’s exact test or Student t tests were used for statistical analysis. P ≤ 0.05 were considered to be significant.

## Results

### XRP44X Inhibits Cell Tumour Formation by Xenografts in Nude Mice

**Fig 1:**
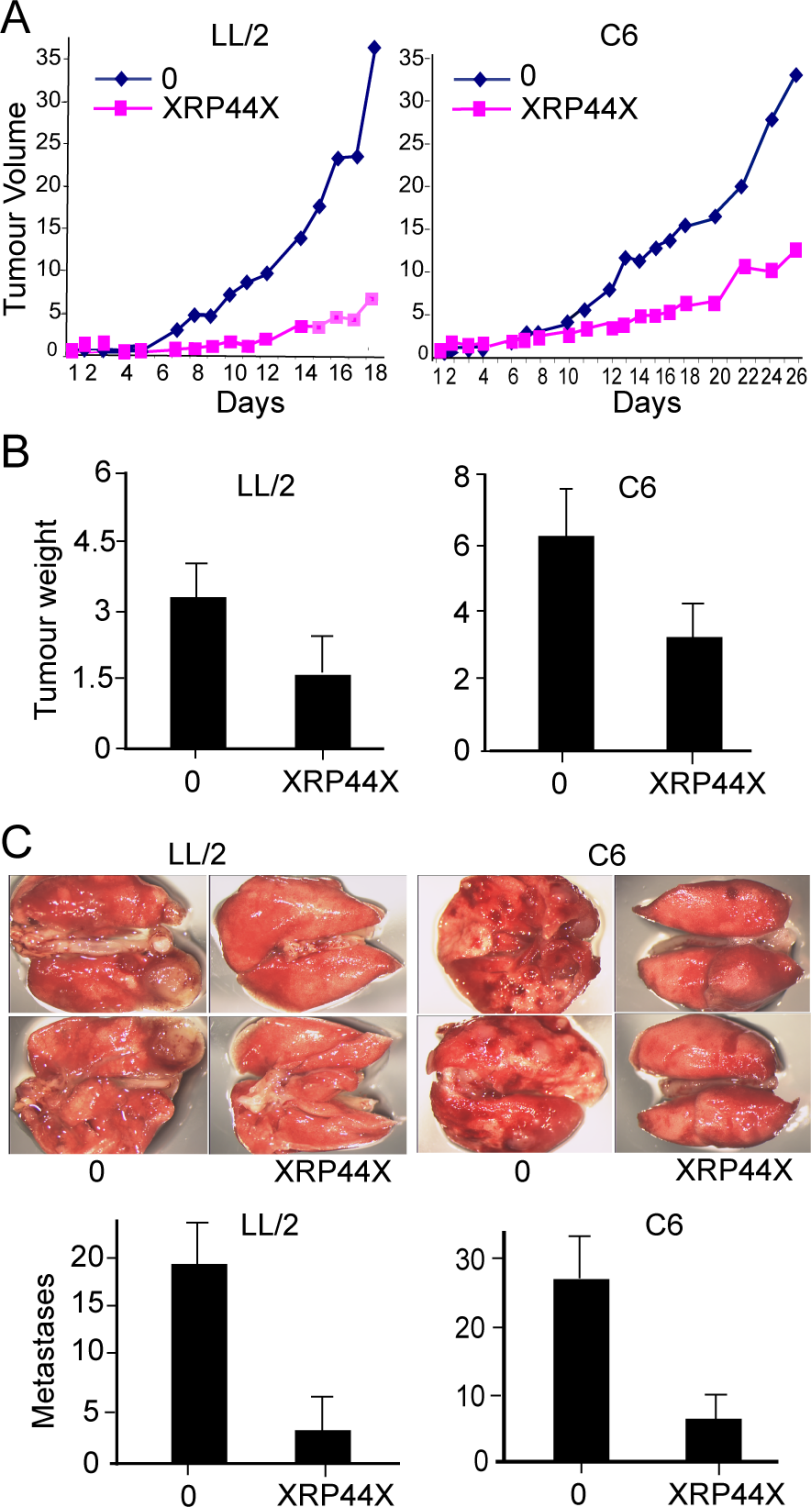
XRP44X inhibits tumour growth and metastasis in nude mice. Cells were injected subcutaneously into nude mice, and after 6 days the mice were treated daily by intraperitoneal injection of XRP44X (1 mg/kg). (A) Tumour volume. One representative result is shown, and similar inhibition was observed in four other mice for each condition and cell line. Comparable results were obtained in three separate experiments. (B) Tumour weight after sacrifice. The average weight and standard deviation for 5 mice per condition is shown (p<0.05). (C) Lung metastases. The lungs were excised after sacrifice, photographed and visible metastases were counted. The averages and standard deviations for 5 lungs per condition are shown.

In order to study the effects of XRP44X on tumour growth and metastasis in-vivo, two cell lines were used, LLC1 (LL/2) Lewis Lung Carcinoma [37] and C6 glioma [38] cells. We previously reported that XRP44X inhibited FGF-2-induced Elk3 phosphorylation in sevral cell lines [18]. Similarly, we found that XRP44X clearly inhibited FGF-2-induced Elk3 phosphorylation in both C6 (S1A Fig) and LL/2 (LLC1) (S1B Fig) (compare FGF-2 induced XRP44X (lanes 4, 5) and U0126 (lanes 1)] treated with FGF-2 induced XRP44X untreated (lanes 3) and non-induced non-treated (lanes 2, 6). LL/2 (LLC1) and C6 cells were injected subcutaneously into nude mice, and after 6 days the mice were injected daily in the peritoneum with 1 mg/kg of XRP44X or vehicle alone. Tumour volume was estimated by measuring with callipers (Fig 1A) and by weighing the tumours after the mice were sacrificed (Fig 1B). Metastases were counted by visual examination of the lungs (Fig 1C). XRP44X inhibited the growth of primary tumours formed by both cell lines, as well as the final weight of the tumours at sacrifice. XRP44X also decreased the number of metastases compared to the controls. Similar results were obtained in three separate experiments, in which 5 mice were used for each condition and cell line (data not shown). There were no evident signs of toxicity of XRP44X, taking into account the appearance and general behaviour of the mice during the experiment, and the overall morphology of the major organs after sacrifice (data not shown). These results show that XRP44X decreases the growth of tumours and the formation of metastases in nude mice.

In order to investigate the mechanisms by which XRP44X inhibits tumour growth, cancer-related gene expression levels were determined using RNA isolated from XRP44X and mock treated tumours and ATLAS^TM^ Mouse Cancer 1.2 Arrays. Genes that were up or down regulated in XRP44X treated C6 and LL/2 (LLC1) tumours (≥1.5 or ≤0.67 fold, 203 genes, see S1 Table) were ranked and analysed with the GSEA v2.1.0 Java desktop application (Gene set enrichment analysis–Broad Institute), with the gene matrixes available from the GSEA website (c1 to c7, v5.0). Gene sets with FDR <25%, or nominal p values <5% were considered. The genes were classified into families using the GSEA webtool [39, 40] (S2 Fig). There is a significant negative enrichment for genes with promoter regions (-2 kb, +2kb) containing the consensus binding motif for Elk1 (SCGGAAGY), which is also the consensus binding motif for Elk3, determined by GREAT mSigDB predicted promoter motif analysis of the most significant Elk3 peaks (p<^E-^15) from ChIP-seq data [7]. There is also negative enrichment for genes up-regulated in MEWO cells (melanoma) after 48h of methionine deprivation, and positive enrichment for genes with promoter regions [-2kb,2kb] around transcription start site containing the motif GGGTGGRR which matches annotation for PAX4: paired box gene 4 and genes up-regulated in NHEK cells (normal epidermal keratinocytes) after UVB irradiation. The negative enrichment for genes containing Elk1/Elk3 binding motifs is expected, since XRP44X was isolated in a screen for inhibition of Elk3 transcription-factor activity [18].

### XRP44X Inhibits Tumour Burden in a Prostate Cancer Bone Metastasis Model

**Fig 2:**
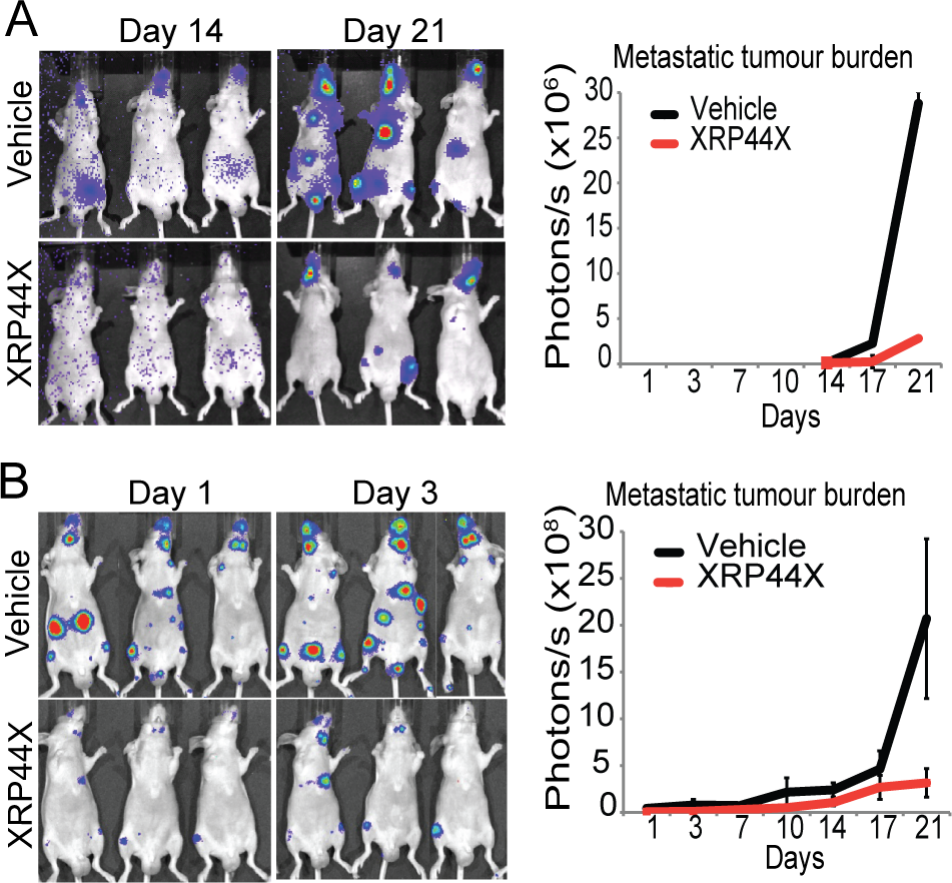
XRP44X decreases tumour burden in a prostate cancer bone metastasis model. (A) Analysis of late stages of metastasis formation. PC-3Mpro4/luc+ cells were inoculated into the left ventricle of the heart of five-week old BALB/c nu/nu male mice. 10 mice were treated daily with 1 mg/kg of XRP44X, starting at day 0, and cells were detected by BMI every 3–4 days, starting at day 14. The graph shows metastatic tumour burden as measured by BLI over the course of the treatment. (B) Analysis of early as well as late stages of metastasis formation. PC-3Mpro4/luc2* cells were inoculated, and the mice were treated as in (A), except that 7 mice were used, and BLI measurements were started at day 1. The error bars represent the standard error of the mean of the BLI signal strength between animals in a group.

The anti-metastatic properties of XRP44X were further explored with a widely used animal model of bone metastasis, in which cancer cells expressing luciferase are inoculated into the left ventricle of immune-deficient mice and metastatic growth is followed by bioluminescence imaging (BLI) [41]. Prostate cancer cells expressing luciferase (PC-3M-Pro4/luc+) were inoculated into 4 week old BALB/c nu/nu animals, the mice were treated daily by intra-peritoneal injections of 1 mg/kg XRP44X or vehicle starting at day 0, (10 per group), and the mice were weighed. Growth of prostate cancer cells was monitored by BLI. After 24 days, the animals were sacrificed and rear limb bones were dissected and visualised to evaluate tumour presence and size. Treatment with XRP44X inhibited metastatic tumour burden, as measured by BLI (Fig 2A). There was no significant difference in the weight of the animals at the end of the experiment, and the decrease at early time points was similar to that observed with Combretastatin A4 (S3 Fig), which has properties similar to XRP44X [18]. These results suggest that XRP44X has limited toxicity, similar to Combretastatin A4 that is used in clinical trials. In order to follow the tumours at earlier stages, a related cell line was used, PC-3M-Pro4/luc2*, that expresses an enhanced firefly luciferase with superior expression [42]. Metastatic tumour burden was found to be decreased by XRP44X at earlier time points, and the decrease became greater at the later time points (Fig 2B), essential confirming and consolidating the results with the other cell line.

To confirm these observations and study the mechanisms further, tibia and fibula from the PC-3Mpro4/luc+ experiment were analysed for the number and size of the tumours, by sectioning and observing every fifth slide after H&E staining. In XRP44X-treated mice, there were fewer sections with metastases, the metastases were of smaller size and were present in fewer animals (S4 Fig). In the vehicle-treated group, two mice had more than one tumour in the bone. Tumours were detected in the tibia, but not in the fibula (data not shown). Overall, these morphological findings confirm the BLI data. In order to detect Elk3, sections were stained with a human Elk3 antibody. There were strong nuclear signals in the metastatic human cells, and not in surrounding mouse tissues, as expected from the specificity of the antibody for human Elk3. The staining was similar for both experimental groups (data not shown). These results show that Elk3 is expressed in the metastases, and, although decreases in Elk3 expression in individual cells of XRP44X treated animals were not evident, there were fewer cells with Elk3 in the treated animals.

### XRP44X Inhibits Tumour Progression in the TRAMP Prostate Cancer Model

**Fig 3:**
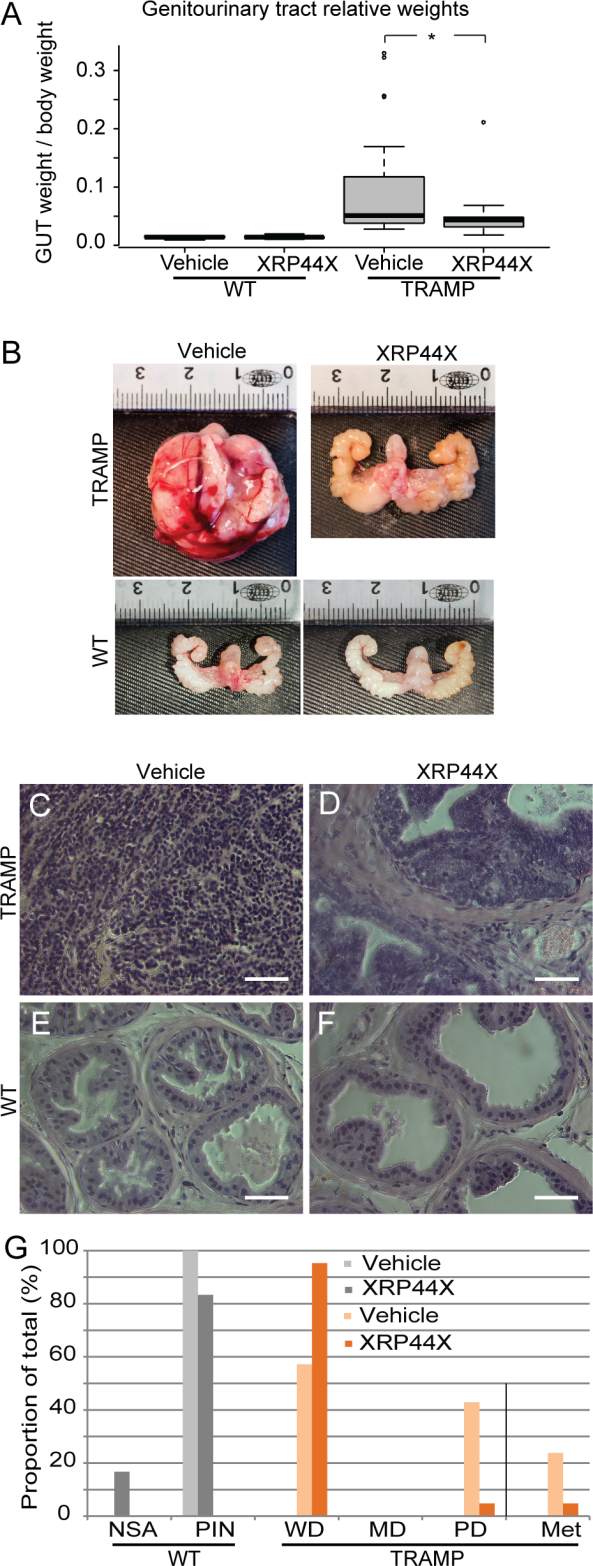
Effect of XRP44X treatment on tumorigenesis in the TRAMP prostate cancer mouse model. Wild type (WT) and TRAMP mice were treated with XRP44X (1 mg/kg) daily, 6 days per week, from 14 to 29 weeks of age. (A, B) Genitourinary tracts (GUT) were taken as a whole (prostate, seminal vesicles, urinary bladder). Their weights relative to body weight are shown in (A) (* P = 0.031), and representative photographs are shown in (B). (C-F) The prostates were sectioned, stained with haematoxylin and eosin and dorso-lateral lobes were graded as described in [28]. Typical sections are shown (see S2 Table for overall statistics). (G) Proportion of wild type and TRAMP mice with the different prostate pathological grades or metastases after treatment with vehicle or XRP44X (NSA—no significant anomalies, PIN—prostatic intraepithelial neoplasia, WD—well differentiated adenocarcinoma, MD—medium differentiated adenocarcinoma, PD–poorly differentiated adenocarcinoma). Scale bars = 50 μm.

The effect of XRP44X on tumour progression in a spontaneous cancer mouse model was analysed using transgenic adenocarcinoma of the mouse prostate (TRAMP) mice, one of the most used and characterised models of prostate cancer progression [24]. An intervention type protocol was used [28], in which WT and heterozygous TRAMP male mice were treated with either XRP44X (1.0 mg/kg) or vehicle (DMSO) by intraperitoneal injection at the same time of day, 6 days per week, from 15 to 29 weeks of age (inclusive). The mice were euthanized, organs were dissected and weighed, and the prostates were fixed, sectioned and H&E stained to determine pathological grade. The weights of the genitourinary tracts of the XRP44X treated TRAMP mice were found to be significantly decreased compared to the vehicle treated animals (p = 0.031, Student’s t-test between the TRAMP groups; Fig 3A). The genitourinary tracts were in general visibly smaller (see representative photographs, Fig 3B). Pathological grades were determined according to Greenberg et al. [28]. Prostate lesions in the dorsolateral prostate lobes were histologically graded as poorly differentiated (PD) adenocarcinoma (sheets of neoplastic cells with little or no glandular structures) (example Fig 3C), moderately differentiated carcinoma (MD, anaplastic sheets of cells that may contain remnants of glandular architecture), well differentiated (WD) carcinoma (increased quantity of small glands) (Fig 3D), PIN (epithelial stratification and tufting, presence of cribriform structures, elongated nuclei, micropapillary projections) (Fig 3E and 3F), and normal (moderate infolding of the mostly columnar epithelia; no significant abnormalities, NSA). XRP44X significantly decreased the proportion of tumours with the more advanced poorly differentiated grade in the TRAMP group (p = 0.008; Fisher’s exact test), and the proportion of TRAMP mice with metastases Fig 3G; S2 Table). There were no significant effects of XPR44X in the wild type mice. These results show that XRP44X inhibits tumour progression in TRAMP mice.

There were no overt signs of toxicity due to this prolonged treatment with XRP44X. There were no significant changes in the observable health and behaviour of the animals, body weight (S5 Fig), and the appearance and weight of the major organs (S6 Fig). Toxicity was further evaluated using whole blood and plasma. The whole blood analysis included white and red blood cells, haemoglobin, haematocrit, mean corpuscular volume and haemoglobin concentration, neutrophils, lymphocytes, monocytes, eosinophils, large unstained cells, basophils, platelets and mean platelet volume. There were no significant changes in the majority of these measurements, except for lower amounts of monocytes and basophiles in XRP44X-treated mice (S3 Table; vehicle n = 9, XRP44X n = 10, all wild type). The blood plasma measurements included of the levels of albumin, total bilirubin, lactate dehydrogenase, aspartate amino transferase, alanine amino transferase and creatinine. There were no significant changes in these parameters (S4 Table; vehicle n = 12, XRP44X n = 10, all wild type). In summary, amongst these parameters, there were only significant decreases in monocytes (about 25%) and basophils (about 40%).

**Fig 4:**
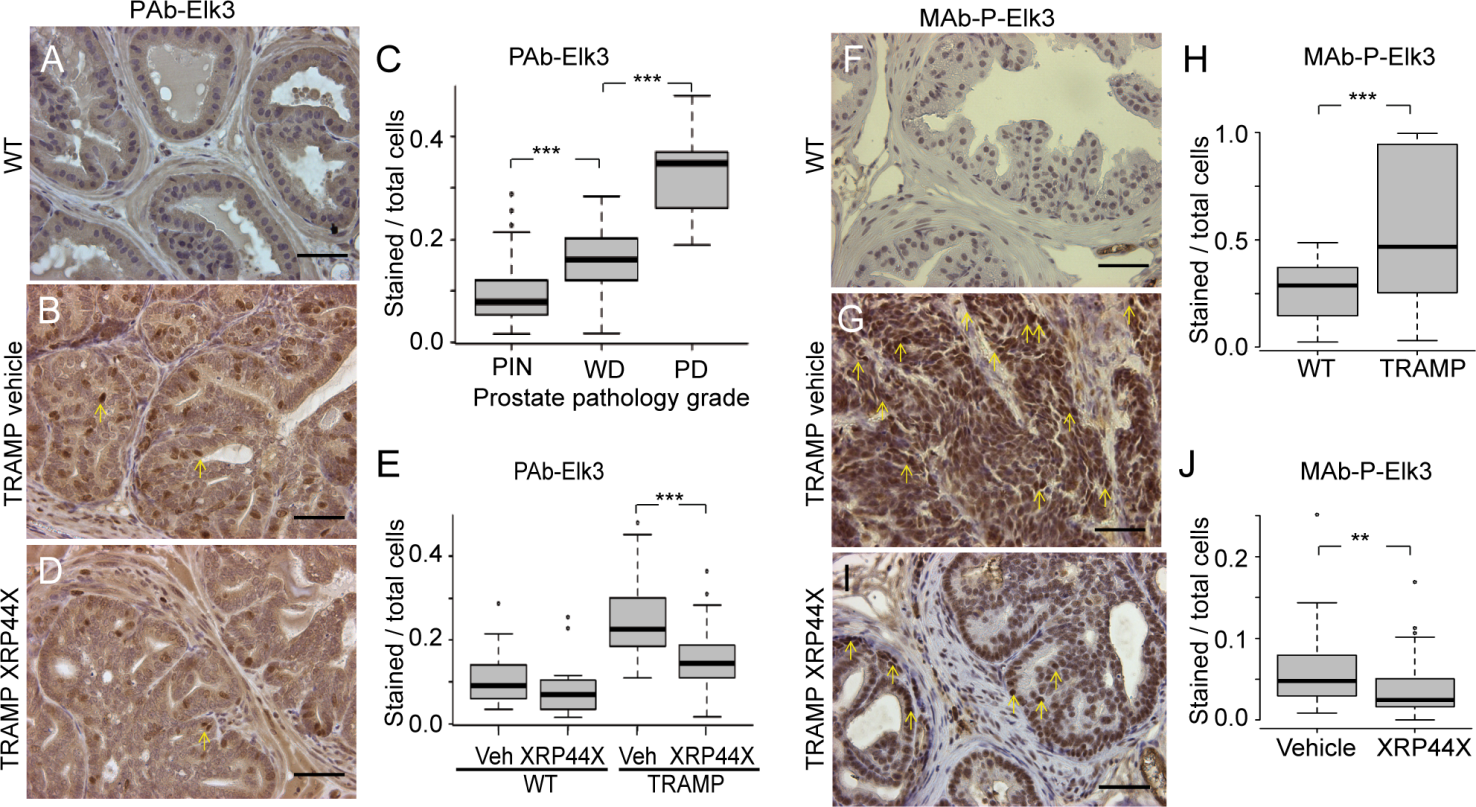
Immunostaining with anti-Elk3 (PAb-95, A-E) and anti-P-Elk3 (MAb-2F3, F-J) of prostates of wild type and TRAMP mice, with and without XRP4X treatment. (A, B, D, F, G & I) Typical stained sections. Scale bars = 50 μm. (C, E, H & J) Boxplots showing the proportion of stained cells in sections of dorsolateral prostates from wild type and TRAMP prostates without or with treatment with XRP44X. Three areas of one slide from each dorsolateral prostate were counted. The numbers of samples were: (C) 12 PIN (prostatic intraepithelial neoplasia); 32 WD (well differentiated tumour); 9 PD (poorly differentiated tumour); (E) 7 WT vehicle (Veh), 6 WT XRP44X, 20 TRAMP vehicle, 21 TRAMP XRP44X; (H) 10 WT and 37 TRAMP; (J) 17 vehicle and 20 XRP44X treated TRAMP prostates (** = P<0.01, *** = P<0.001, Student’s t-test). 29-week-old animals were used.

In order to explore the mechanisms by which XRP44X inhibits tumour progression in TRAMP mice, immunohistochemical (IHC) staining of dorsolateral prostates and tumours was performed with anti-Elk3 (PAb-95) and anti-phospho-Elk3 (MAb-2F3) antibodies. The specificity of the antibodies was authenticated by IHC competition assays with the peptides used for immunisation (PAb-95 and MAb-2F3) and also using pre-immune serum (for PAb-95). Nuclear staing was lost in the presence of the peptides, and was not observed compared to background with the pre-immune serum (S7 Fig). The proportion of cells that stain with the anti-Elk3 antibody (PAb-95) was found to increase with higher pathological grade (Fig 4A–4C; A and B show typical examples of PIN and a WD tumour, respectively, and C the quantification). XRP44X-treated TRAMP tumours were found to have a lower proportion of stained cells than the TRAMP vehicle-treated tumours (Fig 4D and 4E; D shows a representative XRP44X treated WD tumour and E the quantification). Staining with the anti-P-Elk3 antibody (MAb-2F3) increased in TRAMP tumours compared with wild-type prostates (Fig 4F–4H, 4F and 4G are representative examples of PIN and PD tumours, respectively, and H the quantification). The proportion of nuclei that stained strongly for P-Elk3 decreased in the XRP44X treated tumours (Fig 4I and 4J; I is a representative example of a XRP44X treated dorsolateral prostate, and J the quantification). These results indicate that there are changes in immune-reactivity with antibodies directed against Elk3 and P-Elk3, which is consistent with decreased expression and phosphorylation of Elk3 due to XRP44X treatment. However, we cannot exclude, especially in IHC experiments, that other proteins and phosphorylation events are detected by these antibodies.

## Discussion

We report that XRP44X can reduce cancer growth and metastasis in-vivo and has limited toxicity. Initial studies on the mechanisms are consistent with XRP44X targeting pathways associated with Elk3, which is coherent with the screen used to select XRP44X and mechanistic studies with cell lines [18, 22]. These findings encourage further evaluation and development of XRP44X for cancer therapy.

We selected for initial in-vivo characterisation of XRP44X three distinct mouse models of cancer progression and metastasis, subcutaneous xenografts using two highly metastatic cell lines [C6 and LL/2 (LLC1 cells)], intracardiac injection leading to bone metastasis, and prostate cancer progression induced by targeted oncogene expression. These models are reasonably diverse, in terms of: site of tumour growth (subcutaneous, lung, bone and prostate), immunocompetence of the mouse, tumour cell-type (Lewis Lung Carcinoma, glioma, PC3 prostate cancer bone metastasis, and mouse prostate gland) and tumour microenvironment. XRP44X inhibited tumour growth and decreased metastasis in these systems, suggesting that XRP44X is generally effective. All mouse preclinical models have their advantages and disadvantages [23, 43, 44]. Generally, ectopic xenografts of tumour cell lines are facile and reproducible, but lack a native tumour environment and have limited predictive value for most cancers. Some of these disadvantages are overcome in the metastasis to bone model, in that the bone metastasis-derived cells migrate to and proliferate in an osseous environment. Transgenic mouse models such as TRAMP are immunocompetent, generate spontaneous and autochthonous tumours in an appropriate microenvironment and are more predictive in their therapeutic response, but are costly and labour intensive. Evaluations of drugs in mice usually follow a progression of increasing complexity. Potential future directions for the evaluation of XRP44X could include Patient-Derived Xenografts (PDX) and specific Genetically Engineered Mouse Models (GEMM). PDX models are becoming increasing available and characterised [45]. They consist of explanted fragments of tumour tissues in immunosuppressed mice and recapitulate to some extent the human tumour microenvironment. GEMM express a limited selection of the genetic changes that drive human malignancies and mimic the genetic background of human tumour cells [25]. Further evaluation could consider PDX and GEMM driven by mutant Ras, since XRP44X was developed as an inhibitor of mutant Ras / Erk activated transcription.

The treatment regiment was established in initial trial and error experiments in the subcutaneous xenograft models. The choice of conditions was dictated by the solubility of XRP44X in 30% DMSO (final dose 1 mg/kg), the ease of injection (peritoneum) and convenience of the schedule (daily, same time of day). These conditions were subsequently used for the two other models. XRP44X inhibited tumour growth when injected at the beginning (preventative protocol). This included 6 days after subcutaneous injection to allow tumour take, the day of intracardiac injection and after about 14 weeks in the TRAMP model, following puberty (12 weeks) and at about the time at which hyperplasia begins to be observed [46]. Later injection of XRP44X in the xenograft model (after 14 days) also inhibited tumour growth (data not shown). It remains to be seen if XRP44X is more or less effective when different routes, schedules and doses are used.

XRP44X treatment was associated with limited toxicity. There were no overt effects on general health, behaviour, and weights of the body and major organs. There was a transient delay in weight gain in 5-week old nude mice, which was also observed with Combretastatin A4 (CA4) that was injected at a dose that has a similar efficacy (data not shown). XRP44X was compared with CA4 since they have similar effects on microtubules and ERK activity [18, 22]. We found that prolonged treatment of wild type mice with XRP44X decreases monocyte and basophil levels. In contrast, CA4 has been reported to increase granulocytes and decrease neutrophils [47]. The effect of XRP44X are relatively specific to certain types of white blood cell, in contrast to chemotherapeutic agents that usually have more general effects (leukopenia, [48]), including cholchicine that binds to the same site on tubulin [49]. The current results are promising and encourage further evaluation of the safety window.

The effects of XRP44X in-vivo are commensurate with previous observations in-vitro [18, 22]. In the subcutaneous xenograft studies, macroarray and GEA analysis showed that XRP44X down-regulates genes that contain Elk3-like consensus binding motifs in their promoter regions (-2 kb, +2kb), as would be expected from down-regulation of the activity of Elk3 by XRP44X induced inhibition of Ras / Erk phosphorylation. Inhibition of Erk phosphorylation may also inhibit the activity of other Ets factors that bind to similar consensus motifs [2]. Several other gene sets were also found to be enriched, but their connections with Elk3 are more tenuous. Methionine depravation affects genes involved in cell cycle control [50], similar to Elk3 deletion (Gross, 2008 #49}. The positive enrichment for genes with promoters containing Pax4 motifs could be related to the observations that the paralogue Pax5 interacts with Elk3 [51], and aberrant Pax5-Elk3 fusion proteins are found in childhood precursor B-cell acute lymphoblastic leukaemia [52]. The increase in expression of genes that are upregulated by UVB irradiation [53] could be explained by the fact that Jnk is activated by both UVB [54] and XRP44X [22]. Furthermore, the Elk3 repressor is regulated by nuclear export in response to UV [55]. In the intracardiac injection studies, Elk3 was detected in the bone tumours and after XRP44X treatment the volumes of the tumours decreased. The remaining cells continued to express Elk3, perhaps because they were not sufficiently exposed to the compound. In the TRAMP mice, XRP44X decreased the proportion of cells that stained with antibodies raised against Elk3 and phosphorylated Elk3, as would be generally expected from the activities of XRP44X. Dephosphorylation of Elk3 could decrease its stability, as suggested by preliminary observations (unpublished). The effects of XRP44X on gene expression can be expected to be broader than its effects on Elk3, given that it affects Ras / Erk signalling. Interestingly, other chemotherapeutic agents have been shown to affect Ets transcription factor activity. For example, cytosine arabinoside (ARA-C) was identified as an EWS-Fli1 modulator by comparing the transcription-signatures of the compound and the fusion protein [56]. Doxorubicin, Trabectadine, Mithramycin and Midostauin also inhibit the EWS-Fli1 transcriptional signature [57, 58]. In addition, pharmacological inhibitors of Poly(ADP-Ribose) Polymerase inhibit TMPRSS2-ERG activity [59], and Luteolin up-regulates the prostate-derived Ets factor leading to inhibition of cell proliferation and cell invasion in prostate carcinoma cells [60]. Conversely, Ets2 in association with mutant p53 can promote resistance to etoposide [61]. XRP44X, like these other molecules, appear to act at least in part through effects on Ets factor signalling pathways.

In conclusion, we have found that XRP44X inhibits tumour growth and metastasis in-vivo, with limited toxicity. This activity could perhaps be mediated in part by its effects on the Ets transcription factor Elk3. Our study is another example of targeted therapy of a transcription factor through modulation of regulatory and associated pathways and interacting molecules. Our current results encourage further evaluation of XRP44X and related molecules for cancer therapy.

## Supporting Information

S1 Fig XRP44X inhibits FGF-2 induced Elk3 phosphorylation in C6 and LL/2 (LLC1) cells lines in-vitro. C6 (A) and LL/2 (LLC1) (B) cells were plated in 6 well plates and after 36 hours (at about 90% confluence) were pre-treated with compounds (0.1 μM XRP44X, 1 μM XRP44X, 10 μM U0126) or vehicle (DMSO) for 4 h, and then induced with FGF-2 (20 ng/ml) for 10 min. Extracts were analysed by Western blotting for phosphoryled-Elk3 (MAb-2F3) and GAPDH (loading control, MAb374, mouse anti GAPDH IgG1 clone 6C5 Euromedex). Abbreviations: Comp = compound; Conc = concentration; 44 = XRP44X; MW = molecular weight; K = thousand. A similar result was obtained in a biological repeat experiment.
(PDF)

S2 Fig Gene Set Enrichment Analysis. The ranked list of genes that were up and down regulated in XRP44X treated C6 and LL/2 (LLC1) (203 genes, see S1 Table) was analysed using the GSEA v2.1.0 Java desktop application (Gene set enrichment analysis - Broad Institute), with the available gene matrixes (c1 to c7, v5.0). Gene sets with FDR <25%, or nominal p values <5% were considered. (A) Gene sets with the highest and lowest enrichments scores (ES) were selected. SCGGAAGY_V$ELK1_02: Genes with promoter regions [-2kb, 2kb] around transcription start site containing the motif SCGGAAGY which matches annotation for ELK1: ELK1, member of ETS oncogene family. GGGTGGRR_V$PAX4_03: Genes with promoter regions [-2kb, 2kb] around transcription start site containing the motif GGGTGGRR which matches annotation for PAX4: paired box gene 4. KOKKINAKIS_METHIONINE_DEPRIVATION_48HR_UP: Genes up-regulated in MEWO cells (melanoma) after 48h of methionine deprivation. ENK_UV_RESPONSE_KERATINOCYTE_UP: Genes up-regulated in NHEK cells (normal epidermal keratinocytes) after UVB irradiation. ES: enrichment score. NES: normalized enrichment score. NOM p-value: nominal p value. FDR: false discovery rate. FWER: family wise-error rate. (B-E). Enrichment plots.
(PDF)

S3 Fig Effect of XRP44X treatment on body weight of mice in the bone metastasis model. The mice in the experiment shown in Fig 2A were weighed during the course of the experiment. A similar number of mice were treated with Combretastatin A4 (1 mg/kg).
(PDF)

S4 Fig **Effect of XRP44X on tumour size (A-D) and Elk3 expression (E, F) analysed by IHC in the PC-3 pro4/luc+ prostate cancer bone metastasis model**. Rear limb bones (tibias + fibula) of 7 vehicle and 7 XRP44X treated animals (from the experiment in Fig 2A) were cut into longitudinal sections (5 μM thickness), and every 5^th^ slide was stained with haematoxylin and eosin. The sizes of the tumours were estimated by scoring the size on a scale of 1–5 and summing across the slides with tumour in each animal. There was a statistically significant difference in the total size between the two groups (P = 0.046, one tailed Student’s test). (D) Tumours score (as in C) for individual mice. (E, F) Histo- and immuno-staining with PAb against Elk3 (Sigma Prestige® HPA001600). Adjacent sections were stained with H&E and anti-Elk3 PAb followed by goat-anti-rabbit, and revealed with DAB peroxidase substrate kit. The yellow arrowheads point to tumours, the red arrows point in the direction of bone diaphysis and the orange arrows indicate the plane of the bone growth plate. Scale Bars = 100 μM.
(PDF)

S5 Fig Effect of XRP44X treatment on body weight of TRAMP and wild-type mice. TRAMP mice were treated for 6 days per week with XRP44X (1 mg/kg) from 15 to 29 weeks of age. Body weight was recorded twice a week during the course of treatment (WT vehicle n = 14, WT XRP44X n = 14, TRAMP vehicle n = 16, TRAMP XRP44X n = 20). Body weight change was converted into percentage for convenience.
(PDF)

S6 Fig Effect of XRP44X treatment on the weight of organs of wild-type mice. Mice were treated for 6 days per week with XRP44X (1 mg/kg) from 15 to 29 weeks of age. Organs of animals in the wild type and TRAMP experimental groups were weighed at the end of the experiment (number of mice (n): wt vehicle n = 14, wt XRP44X n = 12, TRAMP vehicle 21, TRAMP XRP44X n = 21). A small decrease in the weight (18%) of the spleen of treated wild-type mice cannot be excluded, given the precision of the technique and the number of animals used. There was no significant decrease of the weight of spleen in the TRAMP group.
(PDF)

S7 Fig **Control experiments for antibody specificity using peptide competition (A-C, G-I) and pre-immune serum (D-F).** Adjascent sections (A-C, D-F, G-I) of 30-week old TRAMP mouse prostates were analysed by IHC with PAb-95 (A-C), pre-immune serum for PAb-95 (D-F) and MAb-2F3 (G-I). (A, G) IHC without added peptide. (B, C, H, I) peptide competitions. The peptides used for immunisation were mixed with the corresponding antibodies at increasing concentrations (B, H, 1 μg/ml; C, I, 100 μg/ml). Pre-immune serum was used at three different dilutions (D, 1/1000; E, 1/2500; F, 1/5000). The magnifications are the same for adjascent sections (A-C, D-F, G-I).
(PDF)

S1 Table Expression changes of cancer related genes in subcutaneous xenografts. Expression changes in tumours from LL/2 xenografted animals treated with XRP44X for 18 days and C6 xenografted animals treated with XRP44X for 26 days compared to corresponding control tumours, using cancer gene arrays.
(PDF)

S2 Table Effect of XRP44X treatment on pathology grade in the TRAMP prostate cancer mouse model. TRAMP mice were treated for 6 days per week with XRP44X (1 mg/kg) from 15 to 29 weeks of age. Prostates were harvested, sectioned and stained with haematoxylin and eosin. Prostate adenocarcinoma grade was determined as described in Kaplan-Lefko et al. [28] on one prostate tissue slide per mouse that had a representative amount of dorsolateral prostate. (NSA–no significant anomalies, PIN—prostatic intraepithelial neoplasia, WD–well differentiated adenocarcinoma, MD–medium differentiated adenocarcinoma, PD–poorly differentiated adenocarcinoma. P = 0.008 between TRAMP groups, Fisher’s exact test).
(PDF)

S3 Table Effect of XRP44X treatment on whole blood indicators. TRAMP mice were treated for 6 days per week with XRP44X (1 mg/kg) from 15 to 29 weeks of age. Blood was taken 4–5 days before the end of the treatment from periorbital sinus and analysed on the same day. The animals were not treated 36 hours before the blood sampling. The table shows averages from raw values, their standard deviations and Student’s t-test (vehicle n = 9, XRP44X n = 10, all wild type). Abbreviations: WBC—white blood cells, RBC—red blood cells, HGB—haemoglobin, HCT—haematocrit, MCV—mean corpuscular volume, MCH—mean corpuscular haemoglobin, MCHC—mean corpuscular haemoglobin concentration, NEUTRO—neutrophils, LYMPHO—lymphocytes, MONO—monocytes, EOSINO—eosinophils, LUC—large unstained cells, BASO—basophils, PLT—platelets, MPV—mean platelet volume, st. dev.–standard deviation, T-TEST–Student’s t-test. Whole blood and serum analyses were performed by the ICS metabolic exploration platform (References: IP00005077, IP0000004829, IP0000004857, IP0000004895, IP0000004915, IP0000004949, IP0000005011).
(PDF)

S4 Table Effect of XRP44X treatment on toxicity-related plasma indicators. TRAMP mice were treated for 6 days per week with XRP44X (1 mg/kg) from 15 to 29 weeks of age. Whole blood was taken by cardiac puncture to separate plasma, which was stored at -20°C before the analysis. The animals were not treated 48 hours before the blood sampling. The table shows averages from raw values, their standard deviations and Student’s t-test (vehicle n = 12, XRP44X n = 10, all wild type). Abbreviations: T. bilirubin—total bilirubin, LDH—lactate dehydrogenase, ASAT—aspartate amino transferase, ALAT—alanine amino transferase, st. dev.–standard deviation, T-TEST–Student’s t-test.
(PDF)
