# Supplementary figures and images for: XRP44X, an Inhibitor of Ras/Erk Activation of the Transcription Factor Elk3, Inhibits Tumour Growth and Metastasis in Mice
Source: PLoS One. 2016 Jul 18;11(7):e0159531. doi: 10.1371/journal.pone.0159531 (PMC4948895; doi:10.1371/journal.pone.0159531)

## A C6

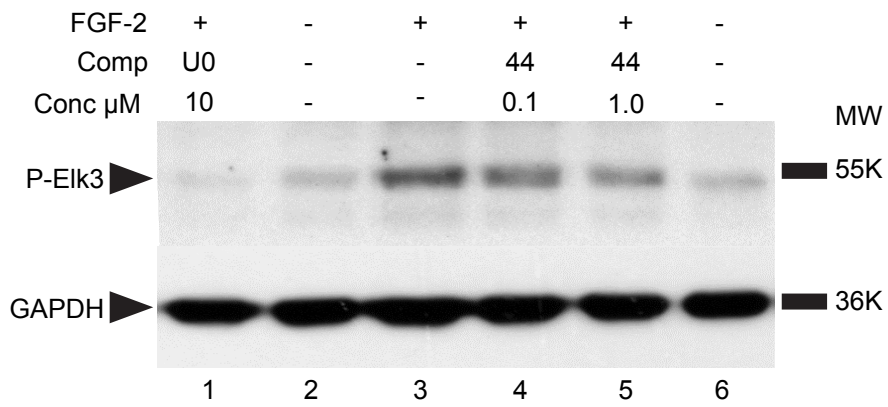

## B LL/2 (LLC1)

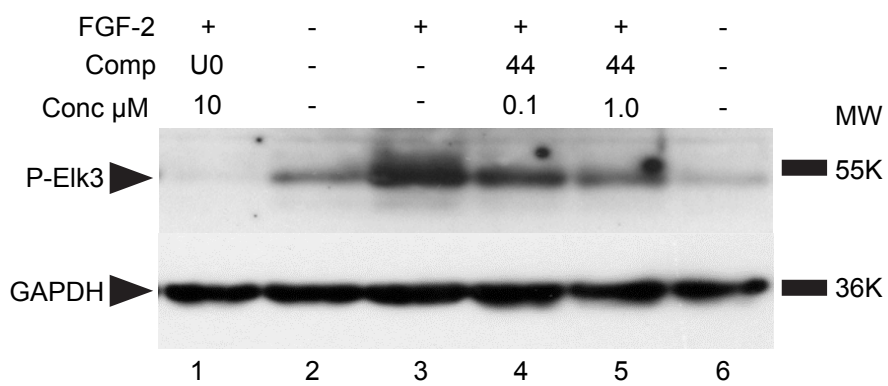

Supplement: S1 Fig — C6 (A) and LL/2 (LLC1) (B) cells were plated in 6 well plates and after 36 hours (at about 90% confluence) were pre-treated with compounds (0.1 μM XRP44X, 1 μM XRP44X, 10 μM U0126) or vehicle (DMSO) for 4 h, and then induced with FGF-2 (20 ng/ml) for 10 min. Extracts were analysed by Western blotting for phosphoryled-Elk3 (MAb-2F3) and GAPDH (loading control, MAb374, mouse anti GAPDH IgG1 clone 6C5 Euromedex). Abbreviations: Comp = compound; Conc = concentration; 44 = XRP44X; MW = molecular weight; K = thousand. A similar result was obtained in a biological repeat experiment. (PDF) [file pone.0159531.s001.pdf]

Body weight with time

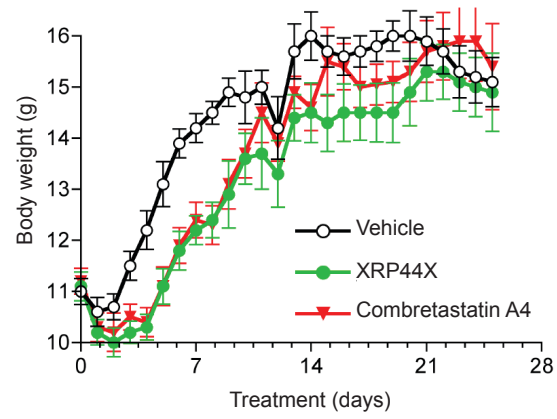

Supplement: S3 Fig — The mice in the experiment shown in Fig 2A were weighed during the course of the experiment. A similar number of mice were treated with Combretastatin A4 (1 mg/kg). (PDF) [file pone.0159531.s003.pdf]

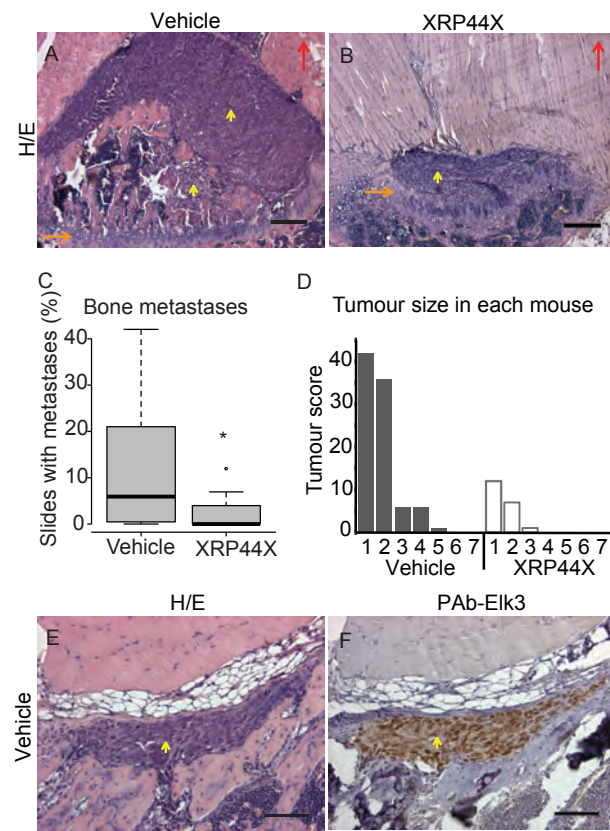

Supplement: S4 Fig — Effect of XRP44X on tumour size (A-D) and Elk3 expression (E, F) analysed by IHC in the PC-3 pro4/luc+ prostate cancer bone metastasis model. Rear limb bones (tibias + fibula) of 7 vehicle and 7 XRP44X treated animals (from the experiment in Fig 2A) were cut into longitudinal sections (5 μM thickness), and every 5th slide was stained with haematoxylin and eosin. The sizes of the tumours were estimated by scoring the size on a scale of 1–5 and summing across the slides with tumour in each animal. There was a statistically significant difference in the total size between the two groups (P = 0.046, one tailed Student’s test). (D) Tumours score (as in C) for individual mice. (E, F) Histo- and immuno-staining with PAb against Elk3 (Sigma Prestige® HPA001600). Adjacent sections were stained with H&E and anti-Elk3 PAb followed by goat-anti-rabbit, and revealed with DAB peroxidase substrate kit. The yellow arrowheads point to tumours, the red arrows point in the direction of bone diaphysis and the orange arrows indicate the plane of the bone growth plate. Scale Bars = 100 μM. (PDF) [file pone.0159531.s004.pdf]

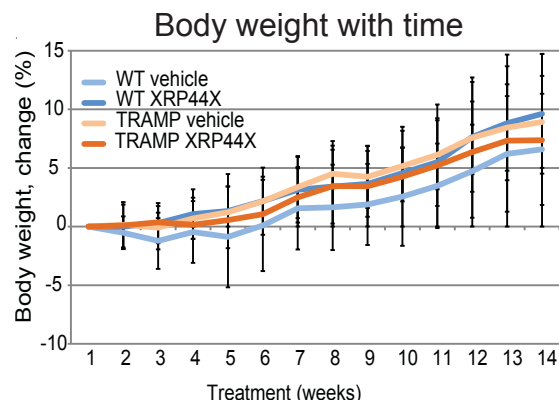

Supplement: S5 Fig — TRAMP mice were treated for 6 days per week with XRP44X (1 mg/kg) from 15 to 29 weeks of age. Body weight was recorded twice a week during the course of treatment (WT vehicle n = 14, WT XRP44X n = 14, TRAMP vehicle n = 16, TRAMP XRP44X n = 20). Body weight change was converted into percentage for convenience. (PDF) [file pone.0159531.s005.pdf]

Major organs relative weight

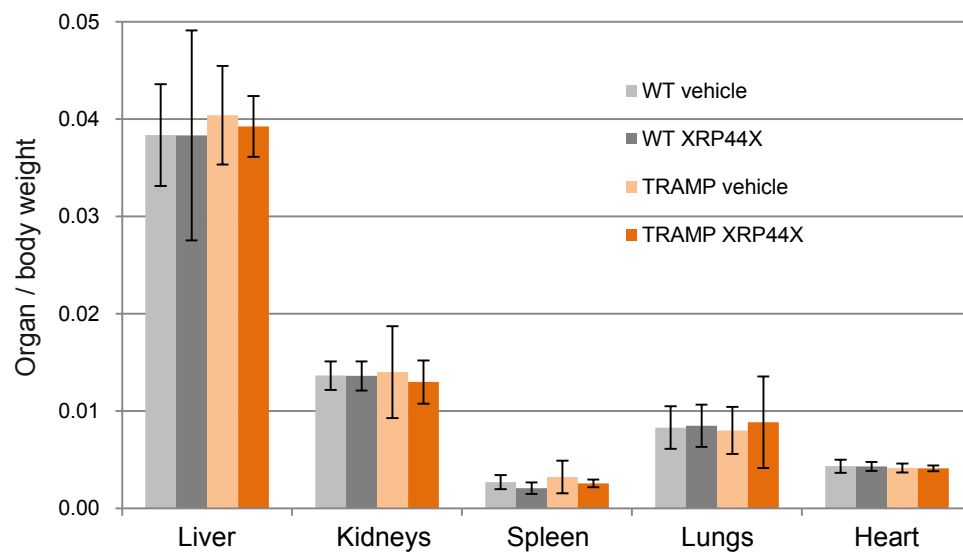

Supplement: S6 Fig — Mice were treated for 6 days per week with XRP44X (1 mg/kg) from 15 to 29 weeks of age. Organs of animals in the wild type and TRAMP experimental groups were weighed at the end of the experiment (number of mice (n): wt vehicle n = 14, wt XRP44X n = 12, TRAMP vehicle 21, TRAMP XRP44X n = 21). A small decrease in the weight (18%) of the spleen of treated wild-type mice cannot be excluded, given the precision of the technique and the number of animals used. There was no significant decrease of the weight of spleen in the TRAMP group. (PDF) [file pone.0159531.s006.pdf]

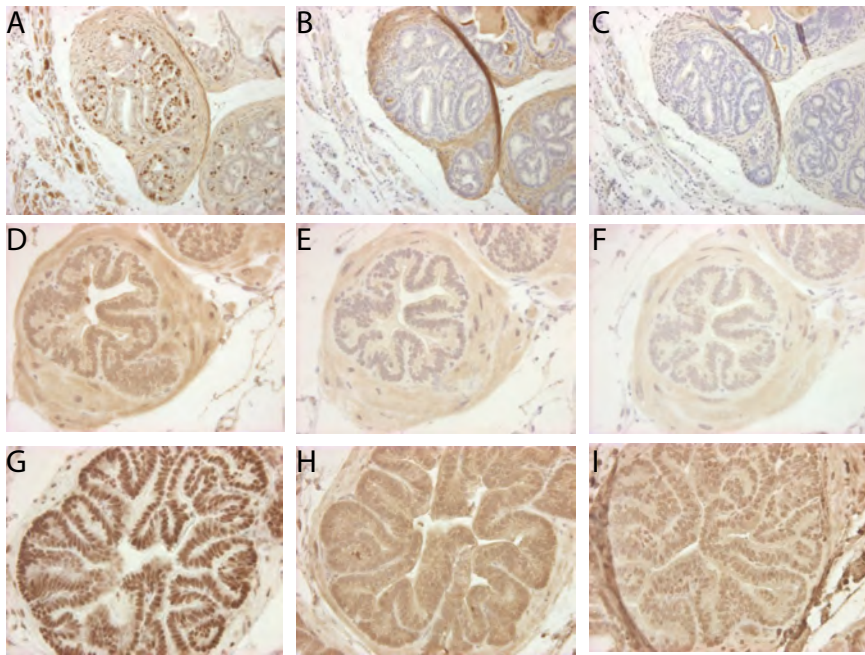

Supplement: S7 Fig — Control experiments for antibody specificity using peptide competition (A-C, G-I) and pre-immune serum (D-F). Adjascent sections (A-C, D-F, G-I) of 30-week old TRAMP mouse prostates were analysed by IHC with PAb-95 (A-C), pre-immune serum for PAb-95 (D-F) and MAb-2F3 (G-I). (A, G) IHC without added peptide. (B, C, H, I) peptide competitions. The peptides used for immunisation were mixed with the corresponding antibodies at increasing concentrations (B, H, 1 μg/ml; C, I, 100 μg/ml). Pre-immune serum was used at three different dilutions (D, 1/1000; E, 1/2500; F, 1/5000). The magnifications are the same for adjascent sections (A-C, D-F, G-I). (PDF) [file pone.0159531.s007.pdf]
