# Supplementary material for: XRP44X, an Inhibitor of Ras/Erk Activation of the Transcription Factor Elk3, Inhibits Tumour Growth and Metastasis in Mice
Source: PLoS One. 2016 Jul 18;11(7):e0159531. doi: 10.1371/journal.pone.0159531 (PMC4948895; doi:10.1371/journal.pone.0159531)

A

GENE SET NAME	ES	NES	NOM p-val	FDR q-val	FWER p-val
SCGGAAGY_V$ELK1_02	-0.37	-1.57	0.029	0.048	0.012
GGGTGGRR_V$PAX4_03	0.40	1.43	0.040	0.84	0.60
KOKKINAKIS_METHIONINE_DEPRIVATION_48HR_UP	-0.36	-1.57	0.019	0.22	0.09
ENK_UV_RESPONSE_KERATINOCYTE_UP	0.50	1.65	0.004	0.77	0.54

B

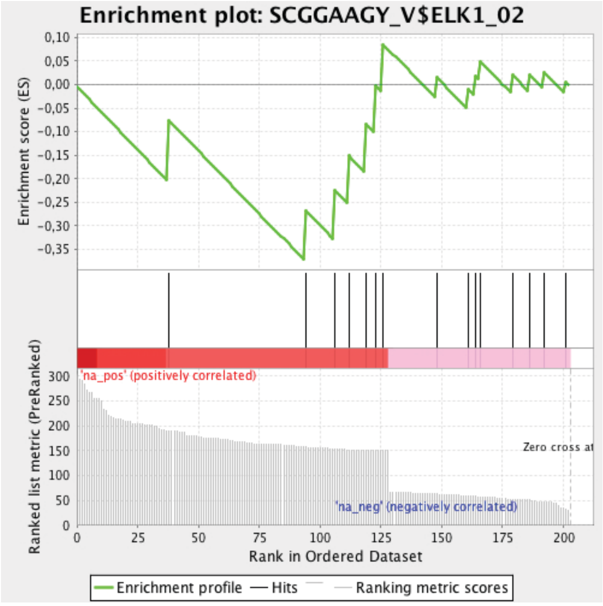

D

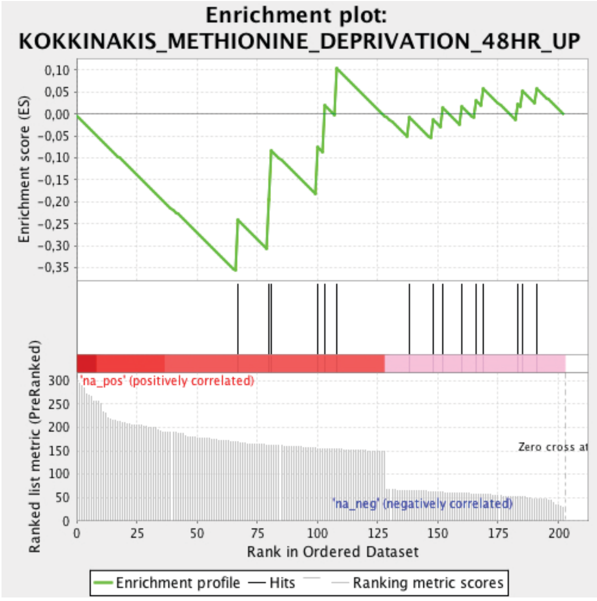

C

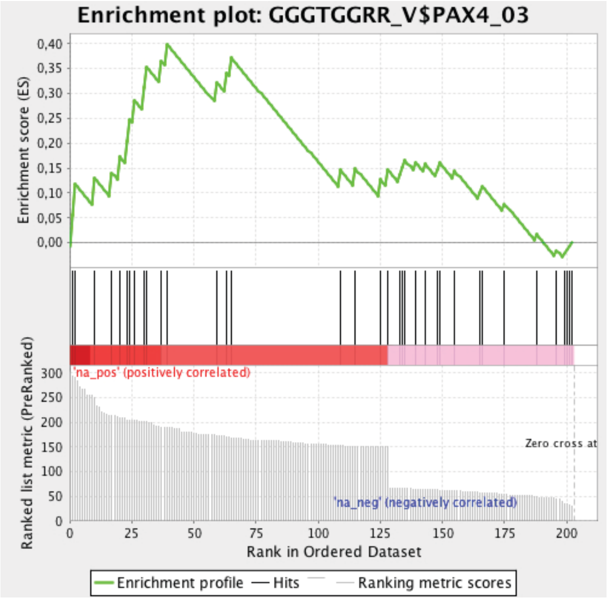

E

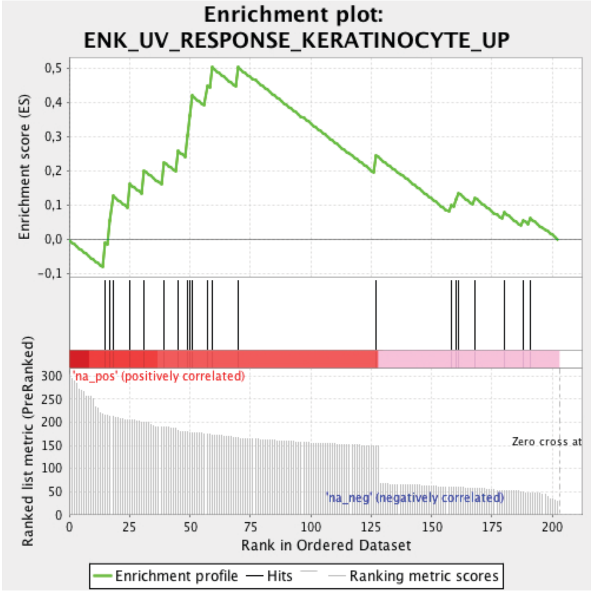

Supplement: S2 Fig — The ranked list of genes that were up and down regulated in XRP44X treated C6 and LL/2 (LLC1) (203 genes, see S1 Table) was analysed using the GSEA v2.1.0 Java desktop application (Gene set enrichment analysis - Broad Institute), with the available gene matrixes (c1 to c7, v5.0). Gene sets with FDR <25%, or nominal p values <5% were considered. (A) Gene sets with the highest and lowest enrichments scores (ES) were selected. SCGGAAGY_V$ELK1_02: Genes with promoter regions [-2kb, 2kb] around transcription start site containing the motif SCGGAAGY which matches annotation for ELK1: ELK1, member of ETS oncogene family. GGGTGGRR_V$PAX4_03: Genes with promoter regions [-2kb, 2kb] around transcription start site containing the motif GGGTGGRR which matches annotation for PAX4: paired box gene 4. KOKKINAKIS_METHIONINE_DEPRIVATION_48HR_UP: Genes up-regulated in MEWO cells (melanoma) after 48h of methionine deprivation. ENK_UV_RESPONSE_KERATINOCYTE_UP: Genes up-regulated in NHEK cells (normal epidermal keratinocytes) after UVB irradiation. ES: enrichment score. NES: normalized enrichment score. NOM p-value: nominal p value. FDR: false discovery rate. FWER: family wise-error rate. (B-E). Enrichment plots. (PDF) [file pone.0159531.s002.pdf]
