# Supplementary material for: XRP44X, an Inhibitor of Ras/Erk Activation of the Transcription Factor Elk3, Inhibits Tumour Growth and Metastasis in Mice
Source: PLoS One. 2016 Jul 18;11(7):e0159531. doi: 10.1371/journal.pone.0159531 (PMC4948895; doi:10.1371/journal.pone.0159531)
Supplement: S1 Table — Expression changes in tumours from LL/2 xenografted animals treated with XRP44X for 18 days and C6 xenografted animals treated with XRP44X for 26 days compared to corresponding control tumours, using cancer gene arrays. (PDF) [file pone.0159531.s008.pdf]

S1 Table. Expression changes of cancer related genes in tumours from LL/2 xenografted animals treated with XRP44X for 18 days and C6 xenografted animals treated with XRP44X for 26 days compared to corresponding control tumours, using cancer gene arrays.

	FOLD	ENTREZ ID	NAME	DESCRIPTION
1	3.01	5788	PTPRC	protein tyrosine phosphatase, receptor type, C
2	2.93	3339	HSPG2	heparan sulfate proteoglycan 2
3	2.90	5331	PLCB3	phospholipase C, beta 3 (phosphatidylinositol-specific)
4	2.83	6708	SPTA1	spectrin, alpha, erythrocytic 1 (elliptocytosis 2)
5	2.72	5793	PTPRG	protein tyrosine phosphatase, receptor type, G
6	2.68	3717	JAK2	Janus kinase 2
7	2.66	7479	WNT8B	wingless-type MMTV integration site family, member 8B
8	2.56	5789	PTPRD	protein tyrosine phosphatase, receptor type, D
9	2.55	6097	RORC	RAR-related orphan receptor C
10	2.55	3627	CXCL10	chemokine (C-X-C motif) ligand 10
11	2.50	8878	SQSTM1	sequestosome 1
12	2.32	345611	IRGM	immunity-related GTPase family, M
13	2.30	59269	HIVEP3	human immunodeficiency virus type I enhancer binding protein 3
14	2.20	8495	PPFIBP2	PTPRF interacting protein, binding protein 2 (liprin beta 2)
15	2.18	4055	LTBR	lymphotoxin beta receptor (TNFR superfamily, member 3)
16	2.15	5696	PSMB8	proteasome (prosome, macropain) subunit, beta type, 8 (large multifunctional peptidase 7)
17	2.14	5599	MAPK8	mitogen-activated protein kinase 8
18	2.13	2783	GNB2	guanine nucleotide binding protein (G protein), beta polypeptide 2
19	2.13	4061	LY6E	lymphocyte antigen 6 complex, locus E
20	2.11	12	SERPINA3	serpin peptidase inhibitor, clade A (alpha-1 antiproteinase, antitrypsin), member 3
21	2.10	4633	MYL2	myosin, light chain 2, regulatory, cardiac, slow
22	2.08	10693	CCT6B	chaperonin containing TCP1, subunit 6B (zeta 2)
23	2.08	1114	CHGB	chromogranin B (secretogranin 1)
24	2.05	558	AXL	AXL receptor tyrosine kinase
25	2.05	655	BMP7	bone morphogenetic protein 7
26	2.05	6354	CCL7	chemokine (C-C motif) ligand 7
27	2.04	3561	IL2RG	interleukin 2 receptor, gamma
28	2.04	56890	MDM1	Mdm1 nuclear protein homolog (mouse)
29	2.02	5465	PPARA	peroxisome proliferator-activated receptor alpha
30	2.02	10728	PTGES3	prostaglandin E synthase 3 (cytosolic)
31	2.01	397	ARHGDIB	Rho GDP dissociation inhibitor (GDI) beta
32	2.01	1839	HBEGF	heparin-binding EGF-like growth factor
33	1.99	4831	NME2	non-metastatic cells 2, protein (NM23B) expressed in
34	1.99	6375	XCL1	chemokine (C motif) ligand 1
35	1.94	7402	UTRN	utrophin
36	1.93	998	CDC42	cell division cycle 42 (GTP binding protein, 25kDa)
37	1.91	6423	SFRP2	secreted frizzled-related protein 2
38	1.90	1398	CRK	v-crk sarcoma virus CT10 oncogene homolog (avian)

39	1.90	3308	HSPA4	heat shock 70kDa protein 4
40	1.90	3695	ITGB7	integrin, beta 7
41	1.90	4043	LRPAP1	low density lipoprotein receptor-related protein associated protein 1
42	1.89	3684	ITGAM	integrin, alpha M (complement component 3 receptor 3 subunit)
43	1.87	1591	CYP24A1	cytochrome P450, family 24, subfamily A, polypeptide 1
44	1.87	2681	GGTA1P	glycoprotein, alpha-galactosyltransferase 1 pseudogene
45	1.87	4683	NBN	nibrin
46	1.81	7040	TGFB1	transforming growth factor, beta 1
47	1.80	22948	CCT5	chaperonin containing TCP1, subunit 5 (epsilon)
48	1.80	908	CCT6A	chaperonin containing TCP1, subunit 6A (zeta 1)
49	1.80	1002	CDH4	cadherin 4, type 1, R-cadherin (retinal)
50	1.80	2919	CXCL1	chemokine (C-X-C motif) ligand 1 (melanoma growth stimulating activity, alpha)
51	1.77	1586	CYP17A1	cytochrome P450, family 17, subfamily A, polypeptide 1
52	1.77	2707	GJB3	gap junction protein, beta 3, 31kDa
53	1.76	71	ACTG1	actin, gamma 1
54	1.76	6347	CCL2	chemokine (C-C motif) ligand 2
55	1.76	1300	COL10A1	collagen, type X, alpha 1
56	1.76	3305	HSPA1L	heat shock 70kDa protein 1-like
57	1.74	2037	EPB41L2	erythrocyte membrane protein band 4.1-like 2
58	1.74	6404	SELPLG	selectin P ligand
59	1.74	7114	TMSB4X	thymosin beta 4, X-linked
60	1.73	1947	EFNB1	ephrin-B1
61	1.73	3460	IFNGR2	interferon gamma receptor 2 (interferon gamma transducer 1)
62	1.72	8728	ADAM19	ADAM metallopeptidase domain 19
63	1.72	4851	NOTCH1	notch 1
64	1.71	3673	ITGA2	integrin, alpha 2 (CD49B, alpha 2 subunit of VLA-2 receptor)
65	1.70	2494	NR5A2	nuclear receptor subfamily 5, group A, member 2
66	1.69	890	CCNA2	cyclin A2
67	1.69	3002	GZMB	granzyme B (granzyme 2, cytotoxic T-lymphocyte-associated serine esterase 1)
68	1.69	3486	IGFBP3	insulin-like growth factor binding protein 3
69	1.68	4288	MKI67	antigen identified by monoclonal antibody Ki-67
70	1.67	1829	DSG2	desmoglein 2
71	1.66	1588	CYP19A1	cytochrome P450, family 19, subfamily A, polypeptide 1
72	1.65	2953	GSTT2	glutathione S-transferase theta 2
73	1.65	3581	IL9R	interleukin 9 receptor
74	1.64	2683	B4GALT1	UDP-Gal:betaGlcNAc beta 1,4- galactosyltransferase, polypeptide 1
75	1.64	10694	CCT8	chaperonin containing TCP1, subunit 8 (theta)
76	1.63	654	BMP6	bone morphogenetic protein 6
77	1.63	284541	CYP4A22	cytochrome P450, family 4, subfamily A, polypeptide 22
78	1.63	6996	TDG	thymine-DNA glycosylase
79	1.63	331	XIAP	X-linked inhibitor of apoptosis
80	1.62	4299	AFF1	AF4/FMR2 family, member 1
81	1.62	317	APAF1	apoptotic peptidase activating factor 1
82	1.62	330	BIRC3	baculoviral IAP repeat containing 3

83	1.62	8851	CDK5R1	cyclin-dependent kinase 5, regulatory subunit 1 (p35)
84	1.62	1466	CSRP2	cysteine and glycine-rich protein 2
85	1.62	5602	MAPK10	mitogen-activated protein kinase 10
86	1.61	22925	PLA2R1	phospholipase A2 receptor 1, 180kDa
87	1.60	1238	CCBP2	chemokine binding protein 2
88	1.60	8738	CRADD	CASP2 and RIPK1 domain containing adaptor with death domain
89	1.60	1396	CRIP1	cysteine-rich protein 1 (intestinal)
90	1.60	8323	FZD6	frizzled family receptor 6
91	1.59	6351	CCL4	chemokine (C-C motif) ligand 4
92	1.59	1944	EFNA3	ephrin-A3
93	1.59	142	PARP1	poly (ADP-ribose) polymerase 1
94	1.58	1572	CYP2F1	cytochrome P450, family 2, subfamily F, polypeptide 1
95	1.57	3316	HSPB2	heat shock 27kDa protein 2
96	1.56	4363	ABCC1	ATP-binding cassette, sub-family C (CFTR/MRP), member 1
97	1.56	4485	MST1	macrophage stimulating 1 (hepatocyte growth factor-like)
98	1.56	4830	NME1	non-metastatic cells 1, protein (NM23A) expressed in
99	1.56	8737	RIPK1	receptor (TNFRSF)-interacting serine-threonine kinase 1
100	1.56	4735	SEPT2	septin 2
101	1.55	993	CDC25A	cell division cycle 25 homolog A (S. pombe)
102	1.55	3001	GZMA	granzyme A (granzyme 1, cytotoxic T-lymphocyte-associated serine esterase 3)
103	1.55	3265	HRAS	v-Ha-ras Harvey rat sarcoma viral oncogene homolog
104	1.55	3320	HSP90AA1	heat shock protein 90kDa alpha (cytosolic), class A member 1
105	1.54	91	ACVR1B	activin A receptor, type IB
106	1.54	1236	CCR7	chemokine (C-C motif) receptor 7
107	1.54	643	CXCR5	chemokine (C-X-C motif) receptor 5
108	1.54	140885	SIRPA	signal-regulatory protein alpha
109	1.53	2697	GJA1	gap junction protein, alpha 1, 43kDa
110	1.53	27040	LAT	linker for activation of T cells
111	1.53	5813	PURA	purine-rich element binding protein A
112	1.52	1544	CYP1A2	cytochrome P450, family 1, subfamily A, polypeptide 2
113	1.52	22919	MAPRE1	microtubule-associated protein, RP/EB family, member 1
114	1.52	6767	ST13	suppression of tumorigenicity 13 (colon carcinoma) (Hsp70 interacting protein)
115	1.51	595	CCND1	cyclin D1
116	1.51	2242	FES	feline sarcoma oncogene
117	1.51	2247	FGF2	fibroblast growth factor 2 (basic)
118	1.51	2814	GP5	glycoprotein V (platelet)
119	1.51	51155	HN1	hematological and neurological expressed 1
120	1.51	4893	NRAS	neuroblastoma RAS viral (v-ras) oncogene homolog
121	1.51	7058	THBS2	thrombospondin 2
122	1.50	10575	CCT4	chaperonin containing TCP1, subunit 4 (delta)
123	1.50	2328	FMO3	flavin containing monooxygenase 3
124	1.50	10808	HSPH1	heat shock 105kDa/110kDa protein 1
125	1.50	3640	INSL3	insulin-like 3 (Leydig cell)
126	1.50	3976	LIF	leukemia inhibitory factor (cholinergic differentiation factor)

127	1.50	4763	NF1	neurofibromin 1
128	1.50	5269	SERPINB6	serpin peptidase inhibitor, clade B (ovalbumin), member 6
129	1.50	8651	SOCS1	suppressor of cytokine signaling 1
1	0.67	116	ADCYAP1	adenylate cyclase activating polypeptide 1 (pituitary)
2	0.67	1571	CYP2E1	cytochrome P450, family 2, subfamily E, polypeptide 1
3	0.67	2335	FN1	fibronectin 1
4	0.67	3702	ITK	IL2-inducible T-cell kinase
5	0.66	3913	LAMB2	laminin, beta 2 (laminin S)
6	0.66	176	ACAN	aggrecan
7	0.66	2260	FGFR1	fibroblast growth factor receptor 1
8	0.66	6383	SDC2	syndecan 2
9	0.66	5447	POR	P450 (cytochrome) oxidoreductase
10	0.65	7042	TGFB2	transforming growth factor, beta 2
11	0.65	9181	ARHGEF2	Rho/Rac guanine nucleotide exchange factor (GEF) 2
12	0.65	919	CD247	CD247 molecule
13	0.65	1943	EFNA2	ephrin-A2
14	0.65	9672	SDC3	syndecan 3
15	0.65	7067	THRA	thyroid hormone receptor, alpha
16	0.65	1031	CDKN2C	cyclin-dependent kinase inhibitor 2C (p18, inhibits CDK4)
17	0.64	5580	PRKCD	protein kinase C, delta
18	0.63	3579	CXCR2	chemokine (C-X-C motif) receptor 2
19	0.63	10019	SH2B3	SH2B adaptor protein 3
20	0.63	7157	TP53	tumor protein p53
21	0.62	652	BMP4	bone morphogenetic protein 4
22	0.62	2322	FLT3	fms-related tyrosine kinase 3
23	0.62	5295	PIK3R1	phosphoinositide-3-kinase, regulatory subunit 1 (alpha)
24	0.62	388	RHOB	ras homolog gene family, member B
25	0.61	1857	DVL3	dishevelled, dsh homolog 3 (Drosophila)
26	0.61	900	CCNG1	cyclin G1
27	0.61	2771	GNAI2	guanine nucleotide binding protein (G protein), alpha inhibiting activity polypeptide 2
28	0.61	90990	KIFC2	kinesin family member C2
29	0.61	1326	MAP3K8	mitogen-activated protein kinase kinase kinase 8
30	0.60	1490	CTGF	connective tissue growth factor
31	0.60	1000	CDH2	cadherin 2, type 1, N-cadherin (neuronal)
32	0.60	3315	HSPB1	heat shock 27kDa protein 1
33	0.59	3326	HSP90AB1	heat shock protein 90kDa alpha (cytosolic), class B member 1
34	0.59	2213	FCGR2B	Fc fragment of IgG, low affinity IIb, receptor (CD32)
35	0.59	3603	IL16	interleukin 16
36	0.58	598	BCL2L1	BCL2-like 1
37	0.57	2534	FYN	FYN oncogene related to SRC, FGR, YES
38	0.57	6464	SHC1	SHC (Src homology 2 domain containing) transforming protein 1
39	0.56	102	ADAM10	ADAM metallopeptidase domain 10
40	0.56	5127	CDK16	cyclin-dependent kinase 16
41	0.56	3082	HGF	hepatocyte growth factor (hepapoietin A; scatter factor)

42	0.56	4157	MC1R	melanocortin 1 receptor (alpha melanocyte stimulating hormone receptor)
43	0.56	7185	TRAF1	TNF receptor-associated factor 1
44	0.56	7507	XPA	xeroderma pigmentosum, complementation group A
45	0.55	4916	NTRK3	neurotrophic tyrosine kinase, receptor, type 3
46	0.55	6775	STAT4	signal transducer and activator of transcription 4
47	0.54	4091	SMAD6	SMAD family member 6
48	0.54	3963	LGALS7	lectin, galactoside-binding, soluble, 7
49	0.53	623	BDKRB1	bradykinin receptor B1
50	0.53	915	CD3D	CD3d molecule, delta (CD3-TCR complex)
51	0.53	10188	TNK2	tyrosine kinase, non-receptor, 2
52	0.53	6256	RXRA	retinoid X receptor, alpha
53	0.52	5900	RALGDS	ral guanine nucleotide dissociation stimulator
54	0.52	208	AKT2	v-akt murine thymoma viral oncogene homolog 2
55	0.51	650	BMP2	bone morphogenetic protein 2
56	0.51	2201	FBN2	fibrillin 2
57	0.51	4313	MMP2	matrix metallopeptidase 2 (gelatinase A, 72kDa gelatinase, 72kDa type IV collagenase)
58	0.51	4968	OGG1	8-oxoguanine DNA glycosylase
59	0.49	3484	IGFBP1	insulin-like growth factor binding protein 1
60	0.49	9618	TRAF4	TNF receptor-associated factor 4
61	0.48	7057	THBS1	thrombospondin 1
62	0.48	284	ANGPT1	angiopoietin 1
63	0.48	834	CASP1	caspase 1, apoptosis-related cysteine peptidase (interleukin 1, beta, convertase)
64	0.48	1649	DDIT3	DNA-damage-inducible transcript 3
65	0.48	2249	FGF4	fibroblast growth factor 4
66	0.46	283120	H19	H19, imprinted maternally expressed transcript (non-protein coding)
67	0.46	3055	HCK	hemopoietic cell kinase
68	0.44	1277	COL1A1	collagen, type I, alpha 1
69	0.44	6442	SGCA	sarcoglycan, alpha (50kDa dystrophin-associated glycoprotein)
70	0.40	4257	MGST1	microsomal glutathione S-transferase 1
71	0.35	2039	EPB49	erythrocyte membrane protein band 4.9 (dematin)
72	0.35	3479	IGF1	insulin-like growth factor 1 (somatomedin C)
73	0.33	7075	TIE1	tyrosine kinase with immunoglobulin-like and EGF-like domains 1
74	0.30	6498	SKIL	SKI-like oncogene

Fold = Expression in tumours from XRP44X treated animals divided by control tumours.

Materials and Methods

Tumours were collected following at days 18 (LLC1) and 26 (C6) from mice that were injected with XRP44X or vehicle alone (controls). Median sized tumours were selected for processing. Portions of the tumours were suspended in 3 ml Trizol^{R} (Life Technologies), homogenised with an Ultra-Turrax homogeniser in ice, and total RNA was purified according to the manufacturer's protocol. Total RNA was treated with DNase I (0.1 units/µg RNA) for 30 min at 37°C. RNA degradation was negligible, as determined by the 28/18S ratio following agarose gel electrophoresis. A total of 45 µg for each

sample was incubated with biotinylated oligo(dT) and streptavidin-coated magnetic beads (Atlas Pure Total RNA Labeling system). RNA on the beads was converted to ^{32}P-labeled first-strand cDNA probes with Moloney murine leukemia virus and the appropriate Atlas array gene-specific CDS primer mixtures. Probes were purified by column chromatography (NucleoSpin extraction column; Macherey-Nagel), and specific activity was measured by scintillation counting. The cDNA expression arrays (ATLAS™ Mouse Cancer 1.2 Array and Atlas mouse cDNA expression arrays 588 genes; Clontech) were hybridized for 16 h at 71°C (LLC1) or 62°C (C6) using ExpressHyb (Clontech), washed, and autoradiographed with Kodak Biomax or exposed with Phosphorimager screens and scanned with a Typhoon 8600 (Amersham Biosciences). The array images were processed with Atlas Image 1.5 software (Clontech). Ratios between significant signals for untreated and treated samples were calculated. Ratios were considered to be significant if the changes were equal to or greater than 50% (≥1.5 or ≤0.67).
