# Supplementary material for: XRP44X, an Inhibitor of Ras/Erk Activation of the Transcription Factor Elk3, Inhibits Tumour Growth and Metastasis in Mice
Source: PLoS One. 2016 Jul 18;11(7):e0159531. doi: 10.1371/journal.pone.0159531 (PMC4948895; doi:10.1371/journal.pone.0159531)
Supplement: S2 Table — TRAMP mice were treated for 6 days per week with XRP44X (1 mg/kg) from 15 to 29 weeks of age. Prostates were harvested, sectioned and stained with haematoxylin and eosin. Prostate adenocarcinoma grade was determined as described in Kaplan-Lefko et al. [28] on one prostate tissue slide per mouse that had a representative amount of dorsolateral prostate. (NSA–no significant anomalies, PIN—prostatic intraepithelial neoplasia, WD–well differentiated adenocarcinoma, MD–medium differentiated adenocarcinoma, PD–poorly differentiated adenocarcinoma. P = 0.008 between TRAMP groups, Fisher’s exact test). (PDF) [file pone.0159531.s009.pdf]

S2 Table. Effect of XRP44X treatment on pathology grade in the TRAMP prostate cancer mouse model. TRAMP mice were treated for 6 days per week with XRP44X (1 mg/kg) from 15 to 29 weeks of age. Prostates were harvested, sectioned and stained with haematoxylin and eosin. Prostate adenocarcinoma grade was determined as described in Kaplan-Lefko et al. [118] on one prostate tissue slide per mouse that had a representative amount of dorsolateral prostate. (NSA – no significant anomalies, PIN - prostatic intraepithelial neoplasia, WD – well differentiated adenocarcinoma, MD – medium differentiated adenocarcinoma, PD – poorly differentiated adenocarcinoma. P=0.008 between TRAMP groups, Fisher's exact test).

	**WT**		**TRAMP**	
	**Vehicle**	**XRP44X**	**Vehicle**	**XRP44X**
**NSA**	**0**	**1**	**0**	**0**
**PIN**	**7**	**5**	**0**	**0**
**WD**	**0**	**0**	**12**	**20**
**MD**	**0**	**0**	**0**	**0**
**PD**	**0**	**0**	**9**	**1**
**TOTAL**	**7**	**6**	**21**	**21**
Metastases	***0***	***0***	***5***	***1***
