# Supplementary material for: XRP44X, an Inhibitor of Ras/Erk Activation of the Transcription Factor Elk3, Inhibits Tumour Growth and Metastasis in Mice
Source: PLoS One. 2016 Jul 18;11(7):e0159531. doi: 10.1371/journal.pone.0159531 (PMC4948895; doi:10.1371/journal.pone.0159531)
Supplement: S3 Table — TRAMP mice were treated for 6 days per week with XRP44X (1 mg/kg) from 15 to 29 weeks of age. Blood was taken 4–5 days before the end of the treatment from periorbital sinus and analysed on the same day. The animals were not treated 36 hours before the blood sampling. The table shows averages from raw values, their standard deviations and Student’s t-test (vehicle n = 9, XRP44X n = 10, all wild type). Abbreviations: WBC—white blood cells, RBC—red blood cells, HGB—haemoglobin, HCT—haematocrit, MCV—mean corpuscular volume, MCH—mean corpuscular haemoglobin, MCHC—mean corpuscular haemoglobin concentration, NEUTRO—neutrophils, LYMPHO—lymphocytes, MONO—monocytes, EOSINO—eosinophils, LUC—large unstained cells, BASO—basophils, PLT—platelets, MPV—mean platelet volume, st. dev.–standard deviation, T-TEST–Student’s t-test. Whole blood and serum analyses were performed by the ICS metabolic exploration platform (References: IP00005077, IP0000004829, IP0000004857, IP0000004895, IP0000004915, IP0000004949, IP0000005011). (PDF) [file pone.0159531.s010.pdf]

S3 Table. Effect of XRP44X treatment on whole blood indicators. TRAMP mice were treated for 6 days per week with XRP44X (1 mg/kg) from 15 to 29 weeks of age. Blood was taken 4-5 days before the end of the treatment from periorbital sinus and analysed on the same day. The animals were not treated 36 hours before the blood sampling. The table shows averages from raw values, their standard deviations and Student's t-test (vehicle n=9, XRP44X n=10, all wild type). Abbreviations: WBC - white blood cells, RBC - red blood cells, HGB - haemoglobin, HCT - haematocrit, MCV - mean corpuscular volume, MCH - mean corpuscular haemoglobin, MCHC - mean corpuscular haemoglobin concentration, NEUTRO - neutrophils, LYMPHO - lymphocytes, MONO - monocytes, EOSINO - eosinophils, LUC - large unstained cells, BASO - basophils, PLT - platelets, MPV - mean platelet volume, st. dev. – standard deviation, T-TEST – Student's t-test. Whole blood and serum analyses were performed by the ICS metabolic exploration platform (References: IP00005077, IP0000004829, IP0000004857, IP0000004895, IP0000004915, IP0000004949, IP0000005011).

	**WBC**	**RBC**	**HGB**	**HCT**	**MCV**	**MCH**	**MCHC**
	x10E03 cells/µL	x10E06 cells/µL	g/dL	%	fL	pg	g/dL
Vehicle	9.07	10.79	15.08	53.94	50.08	14.01	27.99
St. dev.	1.78	0.73	0.86	3.16	1.45	0.70	0.83
XRP44X	8.15	10.43	14.34	52.16	50.01	13.74	27.50
St. dev.	1.57	1.20	1.56	5.67	0.93	0.36	0.78
**T-TEST**	**0.25**	**0.45**	**0.22**	**0.41**	**0.91**	**0.32**	**0.20**

	**%NEUTRO**	**%LYMPHO**	**%MONO**	**%EOSINO**	**%LUC**	**%BASO**	**NEUTRO**
	%	%	%	%	%	%	x10E03 cells/µL
Vehicle	13.23	78.81	2.72	4.36	0.73	0.21	1.21
St. dev.	2.41	6.08	0.69	3.57	0.25	0.08	0.31
XRP44X	12.32	80.62	2.00	4.39	0.58	0.12	0.99
St. dev.	2.03	2.67	0.72	1.99	0.24	0.09	0.25
**T-TEST**	**0.39**	**0.43**	**0.04**	**0.98**	**0.20**	**0.03**	**0.11**

	**LYMPHO**	**MONO**	**EOSINO**	**LUC**	**BASO**	**PLT**	**MPV**
	x10E03 cells/µL	x10E03 cells/µL	x10E03 cells/µL	x10E03 cells/µL	x10E03 cells/µL	x10E03 cells/µL	fL
Vehicle	7.20	0.25	0.41	0.06	0.02	942.33	4.54
St. dev.	1.47	0.11	0.34	0.02	0.01	343.30	0.91
XRP44X	6.45	0.16	0.35	0.05	0.01	1039.60	4.16
St. dev.	1.34	0.07	0.19	0.02	0.01	358.95	0.19
**T-TEST**	**0.26**	**0.04**	**0.68**	**0.10**	**0.01**	**0.55**	**0.25**
