# Supplementary material for: XRP44X, an Inhibitor of Ras/Erk Activation of the Transcription Factor Elk3, Inhibits Tumour Growth and Metastasis in Mice
Source: PLoS One. 2016 Jul 18;11(7):e0159531. doi: 10.1371/journal.pone.0159531 (PMC4948895; doi:10.1371/journal.pone.0159531)
Supplement: S4 Table — TRAMP mice were treated for 6 days per week with XRP44X (1 mg/kg) from 15 to 29 weeks of age. Whole blood was taken by cardiac puncture to separate plasma, which was stored at -20°C before the analysis. The animals were not treated 48 hours before the blood sampling. The table shows averages from raw values, their standard deviations and Student’s t-test (vehicle n = 12, XRP44X n = 10, all wild type). Abbreviations: T. bilirubin—total bilirubin, LDH—lactate dehydrogenase, ASAT—aspartate amino transferase, ALAT—alanine amino transferase, st. dev.–standard deviation, T-TEST–Student’s t-test. (PDF) [file pone.0159531.s011.pdf]

S4 Table. Effect of XRP44X treatment on toxicity-related plasma indicators. TRAMP mice were treated for 6 days per week with XRP44X (1 mg/kg) from 15 to 29 weeks of age. Whole blood was taken by cardiac puncture to separate plasma, which was stored at -20^{0}C before the analysis. The animals were not treated 48 hours before the blood sampling. The table shows averages from raw values, their standard deviations and Student's t-test (vehicle n=12, XRP44X n=10, all wild type). Abbreviations: T. bilirubin - total bilirubin, LDH - lactate dehydrogenase, ASAT - aspartate amino transferase, ALAT - alanine amino transferase, st. dev. – standard deviation, T-TEST – Student's t-test.

	**Albumin** g/l	**T. bilirubin** µmol/l	**LDH** U/l	**ASAT** U/l	**ALAT** U/l	**Creatinine** µmol/l
Vehicle	30.7	2.8	337.4	80.7	22.4	9.3
St.dev.	2.5	1.0	83.9	26.9	6.5	2.6
XRP44X	32.2	2.3	328.3	101.1	23.3	7.6
St.dev.	1.0	0.2	80.9	47.8	6.6	1.4
**T-TEST**	**0.09**	**0.11**	**0.81**	**0.25**	**0.75**	**0.10**
